# Protected complex percutaneous coronary intervention and transcatheter aortic valve replacement using extracorporeal membrane oxygenation in a high-risk frail patient: a case report

**DOI:** 10.1186/s13256-020-02474-x

**Published:** 2020-09-23

**Authors:** Lukasz Kmiec, Andreas Holzamer, Marcus Fischer, Kurt Debl, Matthäus Zerdzitzki, Christof Schmid, Lars Maier, Michael Hilker, Samuel Sossalla

**Affiliations:** 1 Department of Cardiothoracic Surgery, University Medical Center Regensburg, Franz-Josef-Strauß-Allee 11, 93053 Regensburg, Germany; 2 Department of Internal Medicine II, University Medical Center Regensburg, Franz-Josef-Strauß-Allee 11, 93053 Regensburg, Germany

**Keywords:** TAVR, PCI, ECMO, vascular closure device, case report

## Abstract

**Background:**

Transcatheter aortic valve replacement has become a routine procedure for patients with severe symptomatic aortic stenosis at increased surgical risk. Not much is known about using prophylactic support with venoarterial extracorporeal membrane oxygenation in patients undergoing transcatheter aortic valve replacement and eventually concomitant complex percutaneous coronary intervention.

**Case presentation:**

We present a successful procedure of transcatheter aortic valve replacement and high-risk percutaneous coronary intervention utilizing venoarterial extracorporeal membrane oxygenation for hemodynamic support in a very frail 88-year-old Caucasian woman with severe symptomatic aortic stenosis and coronary bypass grafting in the past.

Echocardiography revealed a “low-flow low-gradient” aortic stenosis (mean transvalvular gradient 30 mmHg, aortic valve area 0.4 cm^2^, significant calcification), a left ventricular ejection fraction of 35%, severe mitral regurgitation with moderate stenosis (mean transvalvular gradient 7 mmHg), with a systolic pulmonary artery pressure of 80 mmHg. Moreover, pre-interventional coronary angiography exposed a severe left main ostial stenosis and sequential subtotal heavily calcified stenosis of the left anterior descending artery . Computed tomographic angiography showed no heavy tortuosity but moderate calcification of the iliofemoral arteries.

The procedure was performed under general anesthesia in our hybrid operating room. Extracorporeal membrane oxygenation was established by left femoral percutaneous cannulation using a 21-Fr venous and 15-Fr arterial cannula. Subsequently, complex percutaneous coronary intervention with implantation of two drug-eluting stents from the left main into the left anterior descending artery was performed via a right femoral arterial 7F sheath. Thereafter, a 23-mm Sapien 3 aortic valve prosthesis (Edwards, Irvine, CA, USA) was implanted via right femoral artery in the usual manner, whereby the arterial pigtail catheter for marking the aortic annulus during transcatheter aortic valve replacement was inserted over a Check-Flo® Hemostasis Assembly (Cook Medical, Bloomington, IN, USA) on a Y-adapter via the arterial extracorporeal membrane oxygenation cannula. After extracorporeal membrane oxygenation decannulation, vascular closure was easily performed using the MANTA vascular closure device in order to reduce procedural time and risk of access site complications.

**Conclusions:**

In summary, we demonstrate the feasibility of elective prophylactic extracorporeal membrane oxygenation implementation in selected very high-risk and frail patients undergoing transcatheter aortic valve replacement and percutaneous coronary intervention in order to avoid intraprocedural complications.

## Background

Transcatheter aortic valve replacement (TAVR) has become a routine procedure for patients with severe symptomatic aortic stenosis at increased surgical risk. Nevertheless, even this minimal invasive therapy approach may result in perioperative hemodynamic instability, especially in high-risk patients undergoing complex procedures combining TAVR and percutaneous coronary intervention (PCI). In these cases, an elective prophylactic extracorporeal membrane oxygenation (ECMO) implantation at experienced centers can be discussed in order to minimize the perioperative risk. Hence, this strategy may extend the actual “therapeutic horizon” of TAVR and offer a realistic chance for patients being excluded for any therapy so far.

## Case presentation

**Fig. 1:**
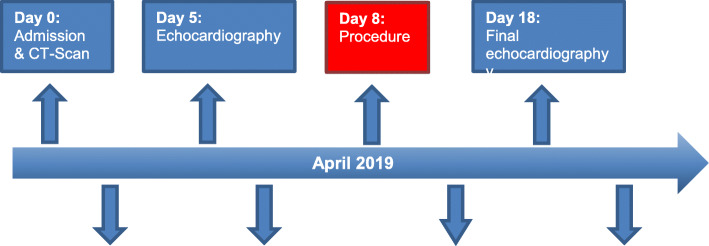
Clinical timeline of the case report

**Fig. 2:**
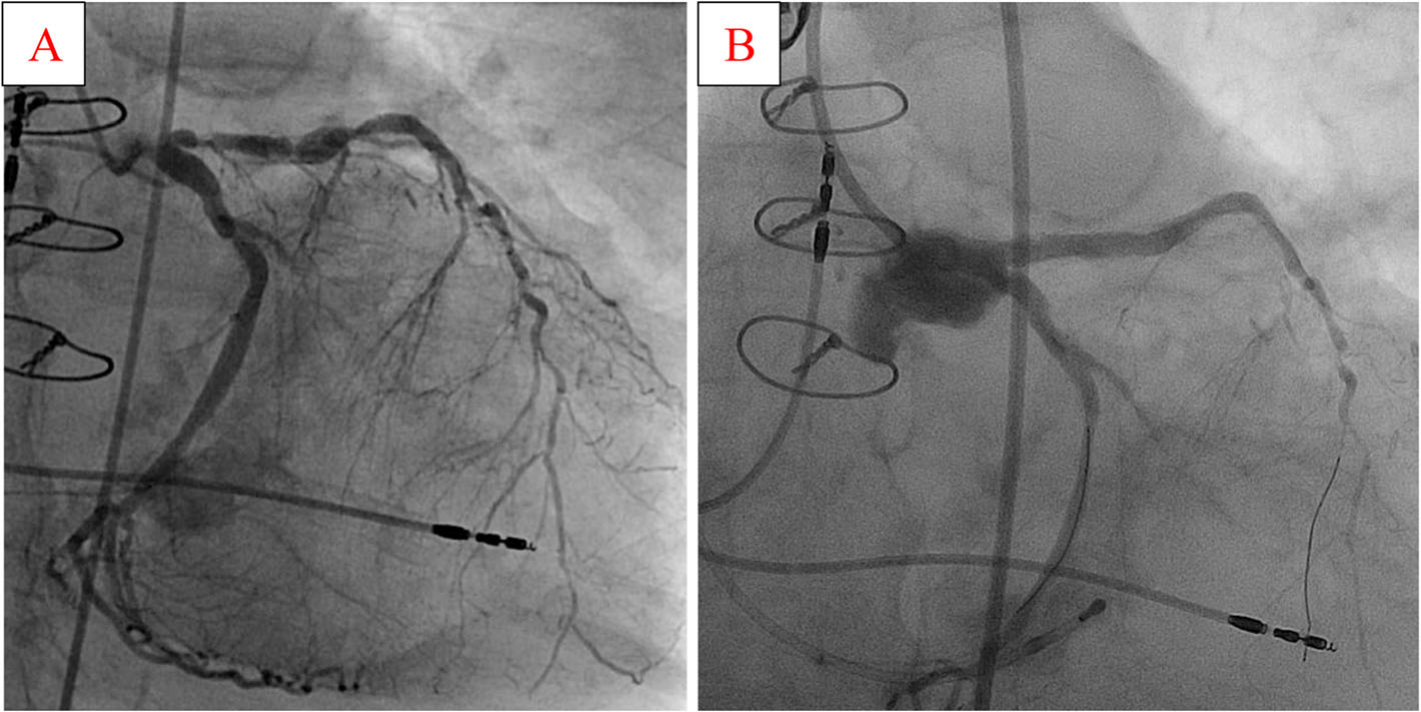
Preprocedural coronary angiography demonstrating the left main ostial stenosis and the sequential subtotal stenosis of the LAD (**a**); the final result after PCI with implantation of two drug-eluting stents from the left main into the LAD (**b**). *LAD* left anterior descending artery, *PCI* percutaneous coronary intervention

In this report, we present the case of a very frail 88-year-old Caucasian woman (height 152 cm, weight 50 kilograms) with a medical history of decompensated heart failure, severe dyspnea (New York Heart Association III), and angina pectoris symptoms (The Canadian Cardiovascular Society II-III) on mild exertion (Fig. 1). Furthermore, syncope or any other acute complaints were denied by the patient during the anamnesis. Echocardiography revealed a “low-flow low-gradient” aortic stenosis (mean transvalvular gradient 30 mmHg, aortic valve area 0.4 cm^2^, significant calcification), a left ventricular ejection fraction of 35%, severe mitral regurgitation with moderate stenosis (mean transvalvular gradient 7 mmHg), with a systolic pulmonary artery pressure of 80 mmHg. Nine years ago, our patient had been surgically treated with coronary bypass grafting. Coronary angiography before TAVR revealed a severe left main (LM) ostial stenosis and sequential subtotal heavily calcified stenosis of the left anterior descending artery (LAD) (Fig. 2a). The left internal thoracic artery to LAD was occluded, and patency of a venous graft to the left circumflex (LCX, OM1) artery could be demonstrated. Her calculated logistic EuroScore I was 59.51% and the Syntax score 56.

Computed tomographic angiography showed no heavy tortuosity but moderate calcification of the iliofemoral arteries. The aortic annular area and perimeter measured 331  mm^2^ and 65.2  mm, respectively.

In our local heart team, the indication for protected high-risk PCI and TAVR was confirmed. Owing to concerns regarding potential hemodynamic instability in our critical patient with the combination of complex and significant un-revascularized proximal coronary stenosis, severe aortic stenosis, impaired LV contractility, mitral valve insufficiency and stenosis with severe pulmonary hypertension, and frailty, it was decided to perform a combined procedure utilizing venoarterial extracorporeal membrane oxygenation (VA ECMO) for hemodynamic support.

Our patient received general anesthesia in our hybrid operating room. VA ECMO was established by left femoral percutaneous cannulation using a 21-Fr venous and 15-Fr arterial cannula (Maquet, Rastatt, Germany). Via a right femoral arterial 7F sheath, PCI was performed accordingly. PCI was complicated by heavy calcification of the proximal LAD and the LM. After subsequent predilatations of LAD and LM using many non-compliant balloons two drug-eluting stents were implanted from the LM into the LAD in a provisional kissing balloon technique in LAD and LCX (Fig. 2b). Stent expansion of the LAD could only be achieved by using two OPN balloons (one ruptured) with 45 bar.

**Fig. 3:**
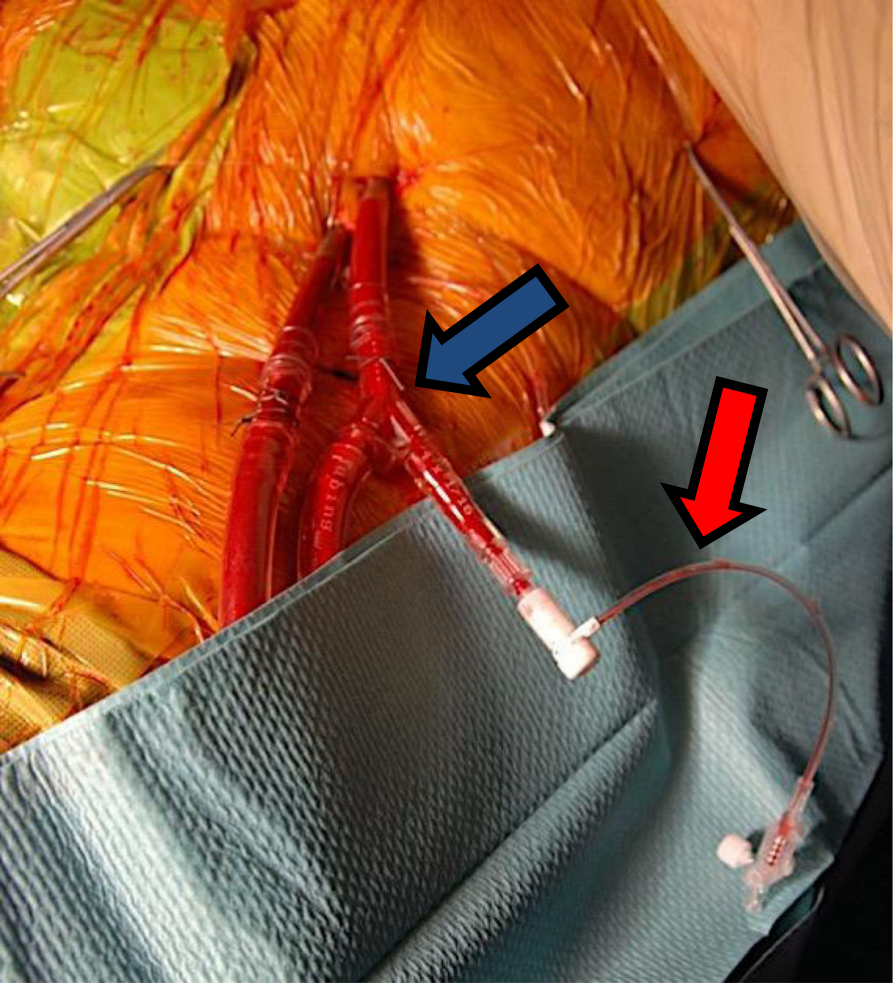
A Check-Flo® Hemostasis Assembly (Cook Medical, Bloomington, IN, USA) (*red arrow*) connected via a Y-adapter (*blue arrow*) to the arterial ECMO cannula using for the insertion of the arterial pigtail catheter. *ECMO* extracorporeal membrane oxygenation

The right femoral 7F sheath was exchanged to the standard expandable TAVR sheath for the Sapien 3 system (Edwards, Irvine, CA, USA). The arterial pigtail catheter that is required for marking the aortic annulus during TAVR was inserted over a Check-Flo® Hemostasis Assembly (Cook Medical, Bloomington, IN, USA) on a Y-adapter via the arterial ECMO cannula (Fig. 3).

**Fig. 4:**
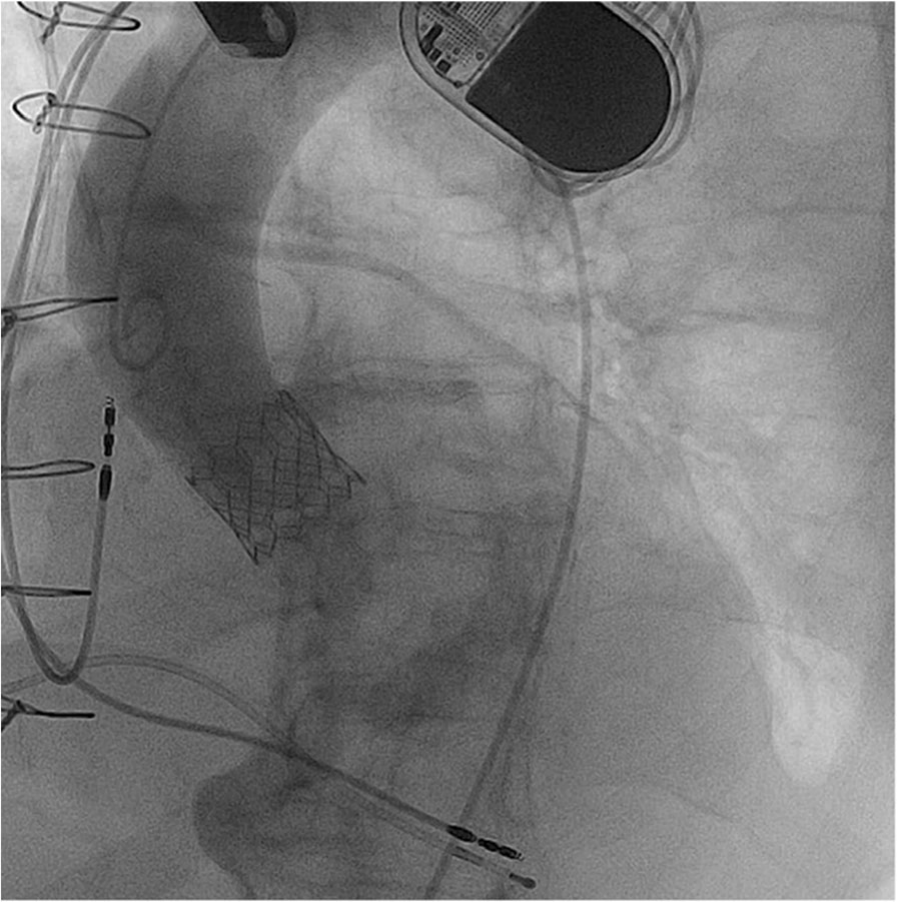
The final angiography of the aortic bulbus after successful transcatheter aortic valve replacement (Sapien 3, 23 mm Edwards, Irvine, CA, USA)

**Fig. 5:**
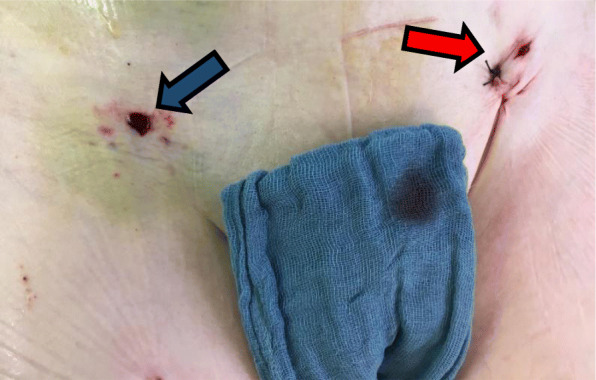
The postoperative result of vascular closure after removing the TAVR sheath (*blue* arrow) and ECMO decannulation (*red arrow*) using the 18F MANTA vascular closure device. *ECMO* extracorporeal membrane oxygenation, *TAVR* transcatheter aortic valve replacement

To reduce the risk of ventricular migration during TAVR, ECMO flow was reduced during valve implantation. A 23-mm Sapien 3 aortic valve prosthesis (Edwards, Irvine, CA, USA) was positioned across the aortic valve. After confirmation of ideal positioning via angiography, the valve was successfully implanted under rapid ventricular pacing. Transesophageal echocardiography and aortography depicted a good result without relevant prosthetic insufficiency (Fig. 4). During LM and LAD PCI, and more pronounced during rapid over-pacing, significant ECMO support was indeed needed. Our patient demonstrated substantial hemodynamic improvement immediately after TAVR. After successful ECMO weaning, we performed decannulation in the operating room using the 18F MANTA vascular closure device (Fig. 5). Hemostasis was immediately achieved, and device-related stenosis or bleeding could be excluded by crossover angiography. The right-sided TAVR introducer sheath was removed, and the arterial puncture site was successfully closed using another MANTA device (Fig. 5). Our patient could be successfully extubated with stable respiration and hemodynamics without need for catecholamines. She was finally transferred to our intensive care unit for further surveillance and could be transferred to our intermediate care unit 5 days later. On day 18, echocardiography revealed an excellent function of the TAVR prosthesis with a mean gradient of 8 mmHg in the absence of any paravalvular regurgitation.

## Discussion

This case demonstrates that even elderly and frail patients undergoing TAVR and complex PCI can be safely treated by temporary prophylactic ECMO support with immediate vascular closure in an elective manner. Not much is known about using prophylactic support with VA ECMO in patients undergoing TAVR and eventually concomitant PCI [1]. We have previously reported that prophylactic ECMO use in selected high-risk TAVR patients was associated with improved procedural success (100%) and 30-day survival to discharge of 100% [2]. A truly different situation is the use of ECMO in rescue cases after TAVR where the risk for complications and increased mortality is much higher [2, 3]. Another study detected comparable findings in a small series of patients undergoing TAVR [3]. Technical success and peri-procedural complications were equivalent to a standard TAVR cohort, suggesting that planned ECMO may be a feasible adjunct in high-risk patients in experienced centers [1, 4]. Therefore, a prophylactic strategy using ECMO may be suitable in situations with severely impaired LV function, expected slow recovery from rapid left ventricular pacing, high vasopressor requirements during general anesthesia or, as in our case, concomitant high-risk PCI [1, 2]. Moreover, an important aspect of prophylactic ECMO is the possibility to employ a safe percutaneous closure device. The novel MANTA system reduces procedural time and device failure in our experience, especially in cases of more than one access sides. Postprocedural hemostasis can be achieved in an entirely percutaneous manner with only rare vascular complications because of the additional cannulation of the femoral vessels with large-bore cannulae in the setting of prophylactic VA ECMO [2].

## Conclusion

In conclusion, instituting prophylactic percutaneous ECMO in selected very high-risk patients undergoing TAVR and complex PCI is a feasible elective strategy in order to avoid intraprocedural complications.

## Data Availability

The data used in this study to support our findings are available upon request from the corresponding author.
